# Genomic insights of high-risk clones of ESBL-producing *Escherichia coli* isolated from community infections and commercial meat in southern Brazil

**DOI:** 10.1038/s41598-022-13197-y

**Published:** 2022-06-07

**Authors:** João Gabriel Material Soncini, Louise Cerdeira, Elder Sano, Vanessa Lumi Koga, Ariane Tiemy Tizura, Zuleica Naomi Tano, Gerson Nakazato, Renata Katsuko Takayama Kobayashi, Caio Augusto Martins Aires, Nilton Lincopan, Eliana Carolina Vespero

**Affiliations:** 1 Laboratory of Clinical Microbiology, Department of Pathology, Clinical and Toxicological Analysis, Health Sciences Center, State University of Londrina, Londrina, Brazil; 2 Department of Infectious Diseases, Central Clinical School, Monash University, Melbourne, Australia; 3 Department of Vector Biology, Liverpool School of Tropical Medicine, Liverpool, UK; 4 Department of Microbiology, Institute of Biomedical Sciences, University of São Paulo, São Paulo, Brazil; 5 Departament of Microbiology, Biological Science Center, State University of Londrina, Londrina, Brazil; 6 Department of Health Science, Health and Biological Science Center, Federal Rural University of Semi-Arid Region, Mossoró, Brazil

**Keywords:** Computational biology and bioinformatics, Microbiology, Molecular biology

## Abstract

During a microbiological and genomic surveillance study conducted to investigate the molecular epidemiology of extended-spectrum β-lactamase (ESBL)-producing *Escherichia coli* from community-acquired urinary tract infections (UTI) and commercial meat samples, in a Brazilian city with a high occurrence of infections by ESBL-producing bacteria, we have identified the presence of CTX-M (-2, -14, -15, -24, -27 and -55)-producing *E. coli* of international clones ST38, ST117, ST131 and ST354. The ST131 was more prevalent in human samples, and worryingly the high-risk ST131-C1-M27 was identified in human infections for the first time. We also detected CTX-M-55-producing *E. coli* ST117 from meat samples (i.e., chicken and pork) and human infections. Moreover, the clinically relevant CTX-M-24-positive *E. coli* ST354 clone was detected for the first time in human samples. In summary, our results highlight a potential of commercialized meat as a reservoir of high-priority *E. coli* lineages in the community, whereas the identification of *E. coli* ST131-C1-M27 indicates that novel pandemic clones have emerged in Brazil, constituting a public health issue.

## Introduction

*Escherichia coli* is a commensal of the human intestinal tract and most warm-blooded mammals, and an important pathogen for humans and animals^1–3^. In humans, urinary tract infections (UTIs) are the second most common bacterial infection managed in primary care, where uropathogenic *E. coli* (UPEC) is responsible for 75% to 95% of the cases^1,4^. The increasing antimicrobial resistance of UPEC has been of concern, with infections caused by antimicrobial-resistant (AMR) bacteria as extended-spectrum β-lactamase (ESBL)-producing *E. coli* representing a significant health care issue, since it compromises the effective treatment, being responsible for a large number of morbidity and mortality^1,3,5^.

Since *E. coli* can act as a large reservoir of resistance genes that directly impact treatment in human and veterinary medicine, the debate over the transmission of multidrug-resistant *E. coli* strains between animals and humans, through numerous pathways, has become increasingly discussed^6,7^. However, the relation between food-producing animals, humans, the environment, and the transmission of these resistant pathogens is not yet fully understood^8,9^.

Specifically, the isolation of ESBL-producing *E. coli* from food-production animals has been increasingly reported worldwide, mostly from chicken meat^1,2^. In this respect, the excessive use of antimicrobials in livestock has promoted the emergence of antimicrobial-resistant pathogens in humans. On the other hand, the consumption of meat, direct contact with colonized animals, or manure spread in the environment have been described as sources for the transmission of AMR pathogens from livestock to humans^3,4^. Besides, antimicrobial resistance gene transfer may occur between different bacterial species in the gut of animals and humans^5^.

The CTX-M type is one of the largest groups of ESBL, and recent studies that addressed the epidemiology of these enzymes in Brazil, show that CTX-M-2, CTX-M-8, CTX-M-9, and CTX-M-15 are predominant variants in this country^6–8^. Many types of CTX-M-producing *E. coli* have been recognized as belonging to specific clones commonly isolated from UTI cases characteristic of a particular region and that are spread throughout the world. Some studies show that CTX-M genotypes of isolates from foods sometimes correspond with locally dominant human clones^9,10^. In this respect, *Escherichia coli* sequence type (ST) 131 is a type of pandemic clone associated with ESBL production^11,12^. Studies describe that the spread of ST131 is stimulated by a specific sub-lineage called clade C or C/H30 and the reason for its successful spread around the world is still not fully understood^13^. Although studies have shown that CTX-M-15 production is strongly involved with the predominant ST131 C2/H30Rx subgroup, the emerging C1 subgroup is related to other CTX-M enzymes, such as CTX-M-14 and CTX-M-27^12,14,15^.

Recently, reports of clade C1-M27 in humans, animals and the environment have been increasing in several countries such as Australia, Canada, France, Germany, Italy, Japan, Netherlands, Spain, Thailand and USA^8,12,14^. The rapid transmission of ST131-C1-M27 becomes a challenge for global health, as it highlights an evolution of ESBLs caused by the ST131 clone and reinforces the complex structure of this clone^12,14–16^.

Considering the impact of the AMR in Brazil, both in human medicine as in livestock, and the need for understanding this panorama, we conducted a next-generation sequencing (NGS)-based analysis, within a One Health perspective, in order to assess national transmission of CTX-M-producing *E. coli* isolated from meat products and human patients*.*

## Results

The results about source, ST, antimicrobial susceptibility and resistome for each of 91 *E. coli* isolates included in this study are quoted in Table 1, more information about the isolates can be seen in "Table 1 complete" in the supplementary material. High rates of resistance to ampicillin (100%), ceftriaxone (87.9%), nalidixic acid (87.9%), cefepime (83.5%), trimethoprim-sulfamethoxazole (82.4%), nitrofurantoin (76.9%), norfloxacin (75.8%) and ciprofloxacin (72.5%) were confirmed, whereas less than half showed resistance to gentamicin (36.3%) and amoxicillin/clavulanate (22%). Only three (3.3%) isolates were resistant to piperacillin-tazobactam and two (2.2%) to amikacin. Some isolates also showed intermediate resistance levels to amoxicillin/clavulanate (28.6%), to piperacillin-tazobactam and gentamicin (4.4%), and to ciprofloxacin and norfloxacin 1.1%).

**Table 1:**
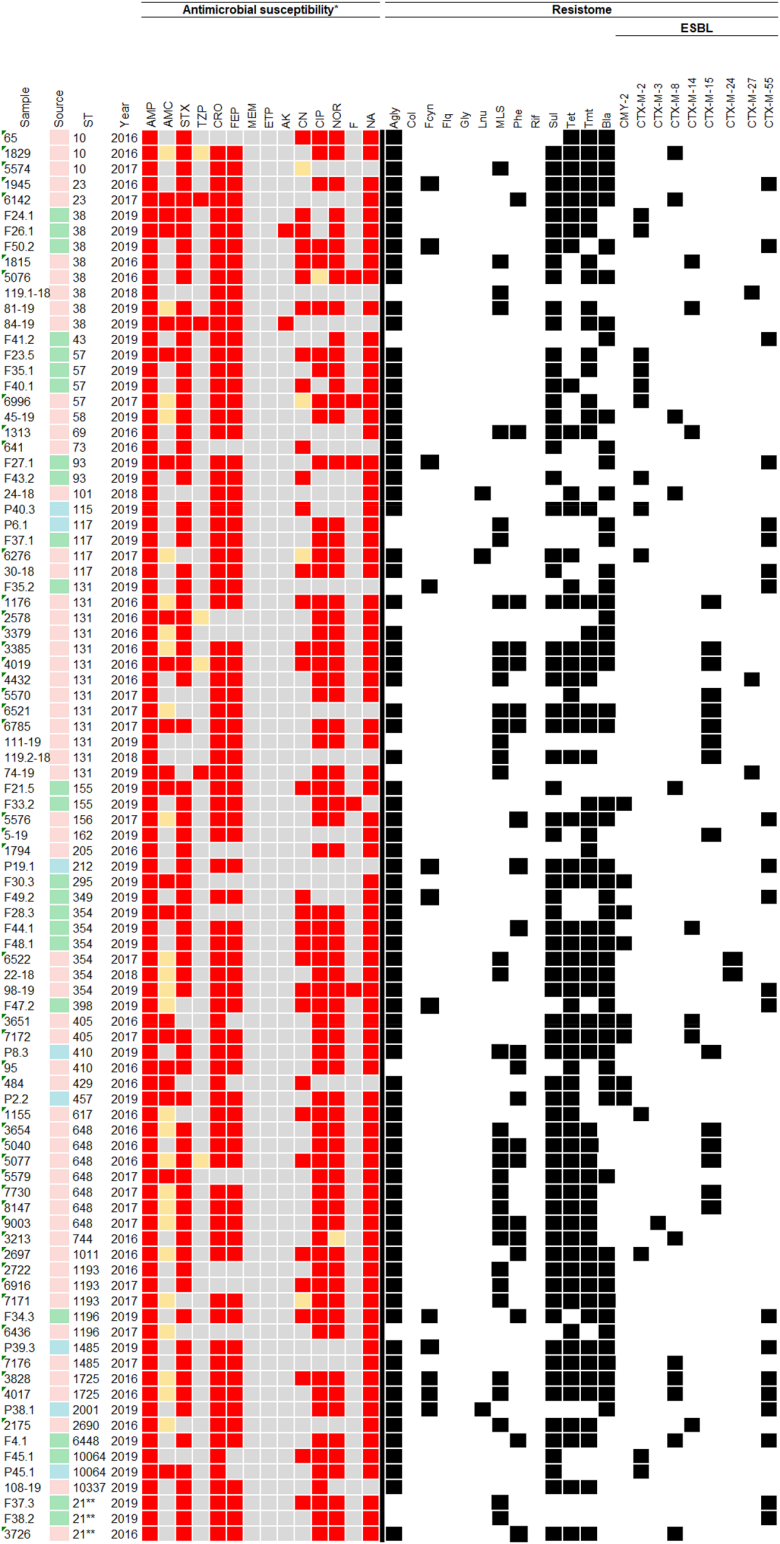
Heatmap shows sample code, its origin, ST, year of isolation, AMR profile and resistome.

Color legend: pink: human; green: chicken meat; blue: pork; red: resistance; orange: intermediate resistance; gray: susceptibility, black: presence confirmed.

Legend of antimicrobials: *AMP* ampicillin, *AMC* amoxicillin-clavulanate, *STX* trimethoprim-sulfamethoxazole, *TZP* piperacillin-tazobactam, *CRO* ceftriaxone, *FEP* cefepime, *MEM* meropenem, *ERP* ertapenem, *AK* amikacin, *CN* gentamicin, *CIP* ciprofloxacin, *NOR* norfloxacin, *F* nitrofurantoin, *NA* nalidixic acid.

Legend of resistome: *Agly* aminoglycosides, *Col* colistin, *Fcyn* fosfomycin, *Flq* fluoroquinolones, *Gly* glycopeptides, *MSL* macrolides, *Phe* phenicols, *Rif* rifampin, *Sul* sulfonamides, *Tet* tetracyclines, *Tmt* trimethoprim, *Bla* β-lactam, *Lnu* lincosamides.

**Escherichia coli scheme sequencetype II (Institut Pasteur).

Genomic analysis predicted 57 genes associated with resistance to aminoglycosides (n = 15), β-lactams (n = 12), trimethoprim (n = 8), phenicols (n = 5), tetracyclines (n = 4), macrolides (n = 4), sulfonamides (n = 3), quinolones (n = 3), lincosamides (n = 2) and fosfomycin (n = 1). For aminoglycosides, the most prevalent genes were *strA* and *strB* (39.6%), followed by the *aadA1* (36.3%). Genes *dfrA17* and *dfrA1* genes, associated with resistance to trimethoprim, were detected in 31 (34.1%) and 13 (14.3%) isolates, respectively. Genes related to phenicols resistance had similar prevalence, being *catB3* (8.8%), *floR* (5.5%), *catA1*(4.4%) and *cmlA1* (4.4%). Concerning to tetracyclines resistance, *tet*(A) (38.5%) and *tet*(B) (27.5%) genes were identified. For macrolides resistance, the *mph*(A) gene (29.7%) was found, whereas sulfonamides resistance *sul1* (56%) and *sul2* (53.8%) genes were predicted. Genes conferring resistance to lincosamides (*lnu*-type) and fosfomycin (*fosA*) were identified in three *E. coli* strains (3.3%).

Resistance to β-lactams was mediated by *bla*_TEM-1A_ (1.5%), *bla*_TEM-1B_ (32.3%), *bla*_OXA-1_ (5.3%), *bla*_CMY-2_ (6.0%) and *bla*_CTX-M_ (54.9%). Among ESBL genes eight variants were identified, with predominance of *bla*_CTX-M-55_ (25.9%), which was identified in *E. coli* strains isolated from chicken meat (n = 11) and pork meat (n = 4), and from human hosts (n = 6). The *bla*_CTX-M-15_ ESBL gene was predominantly in human isolates (n = 14), being further identified in only one pork isolate. On the other hand, the *bla*_CTX-M-2_ gene was also detected in 13 isolates (16%), from chicken meat (n = 7), human (n = 4) and pork meat (n = 2) samples. CTX-M-8 and CTX-M-14 ESBL genes were present in nine and six human isolates, and two and one chicken meat isolates, respectively. CTX-M-24 (n = 2), CTX-M-27 (n = 3) and CTX-M-3 (n = 1) genes were present only in human isolates. The *bla*_CMY-2_ gene was predominantly found in chicken meat samples (n = 4), followed by urine samples (n = 3) and pork meat (n = 1).

In this study, 11 types of plasmid replicons were predicted, which belonged to the Col, IncA/C2, IncB/O/K/Z, IncF, IncI, IncQ, IncR, IncY, IncX, p0111 and IncN families. In human isolates, the most frequent were IncI1[ST-113] (n = 9), IncF[F-: A-: B-] (n = 7), IncF[F1: A2: B20] (n = 5), IncF[F48: A1: B49] (n = 5) and p0111 (n = 5). In chicken meat isolates, IncF[F18: A-: B1] (n = 8), p0111 (n = 7) and IncN[unknown ST] (n = 5) were the most frequent Inc-type plasmid identified. In isolates of pork meat, the most frequent incompatibility groups were IncN[unknown ST] (n = 4) and IncF[F33: A-: B1] (n = 3).

**Figure 1:**
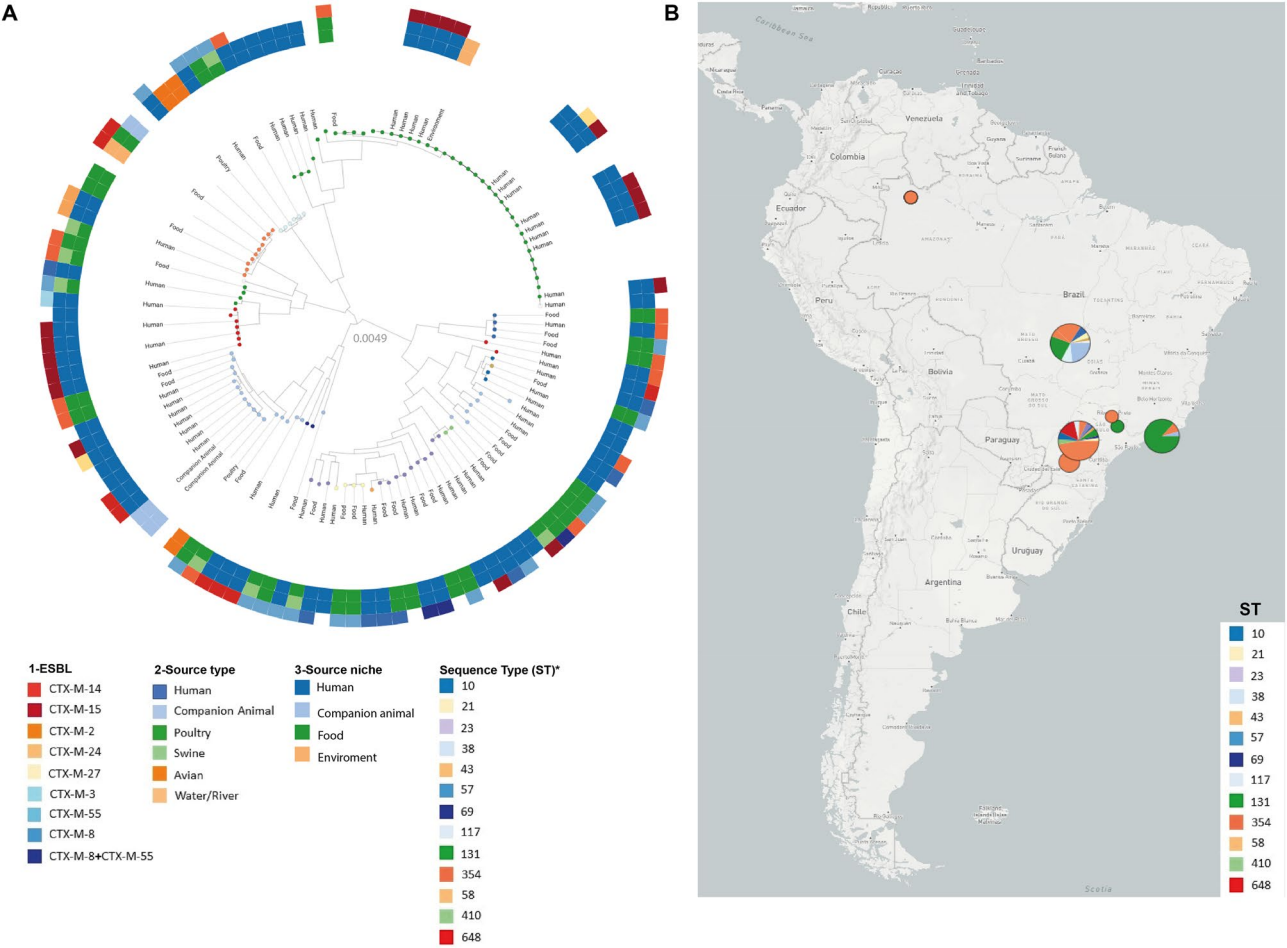
(**A**) *Escherichia coli* Phylogenomic SNP tree with circular heatmap shows (outside-in reading rings): ESBL-gene (first inner ring); source type (second inner circle) and source niches (third inner circle). (**B**) Distribution map of *Escherichia coli* sequence type (ST) in Brazilian regions. Asterisk: bubbles colors in phylogenomic branches.

In total, 40 sequence types (STs) were identified, the most prevalent were ST131 (n = 13), ST38 (n = 8), ST648 (n = 7), and ST354 (n = 6). Some STs were detected in strains isolated from more than one source, confirming clonal dissemination between humans and chicken meat. Lineages of ST38, ST131, ST354, and ST1196 were found in both urine and chicken meat. The ST410 was the only observed in urine (n = 1) and pork (n = 1) strains. The ST117 was identified in samples collected from the three sources investigated, with two strains being isolated from urine, and one from chicken meat and pork, respectively. The clonal relatedness among isolates characterized in this study, and their distribution in Brazil, is shown in Fig. 1A,B. Additionally, it is possible to observe that Brazilian isolates of ST131 clustered with isolates from United Kingdom (https://microreact.org/project/2mKg54AHdWj5xdJ5VFejY8). All information and genes detected in this study are quoted in supplementary material (Table 1) (https://doi.org/10.6084/m9.figshare.17108402.v1).

Some important associations were observed, such as one *E. coli* ST38 isolate producing CTX-M-27 and carrying the IncF[F2:A-:B10] plasmid, and *E. coli* ST131 producing CTX-M-15 carrying various IncF-type plasmids (i.e., IncF[F2:A-:B1], IncF[F2:A1:B-], IncF[F31:A4:B1], IncF[F36:A4:B1] and IncF[F51:A1:B-]). Other important findings are the identification of CTX-M-27 *E. coli* ST131 carrying plasmids IncF[F1:A2:B20], and the co-production of CTX-M-8 and CTX-M-55 by *E. coli* ST1725 and ST6448 harboring IncF[F24:A-:B73], IncF[F33:A-:B1] and/or IncI1[ST-113] plasmids.

**Figure 2:**
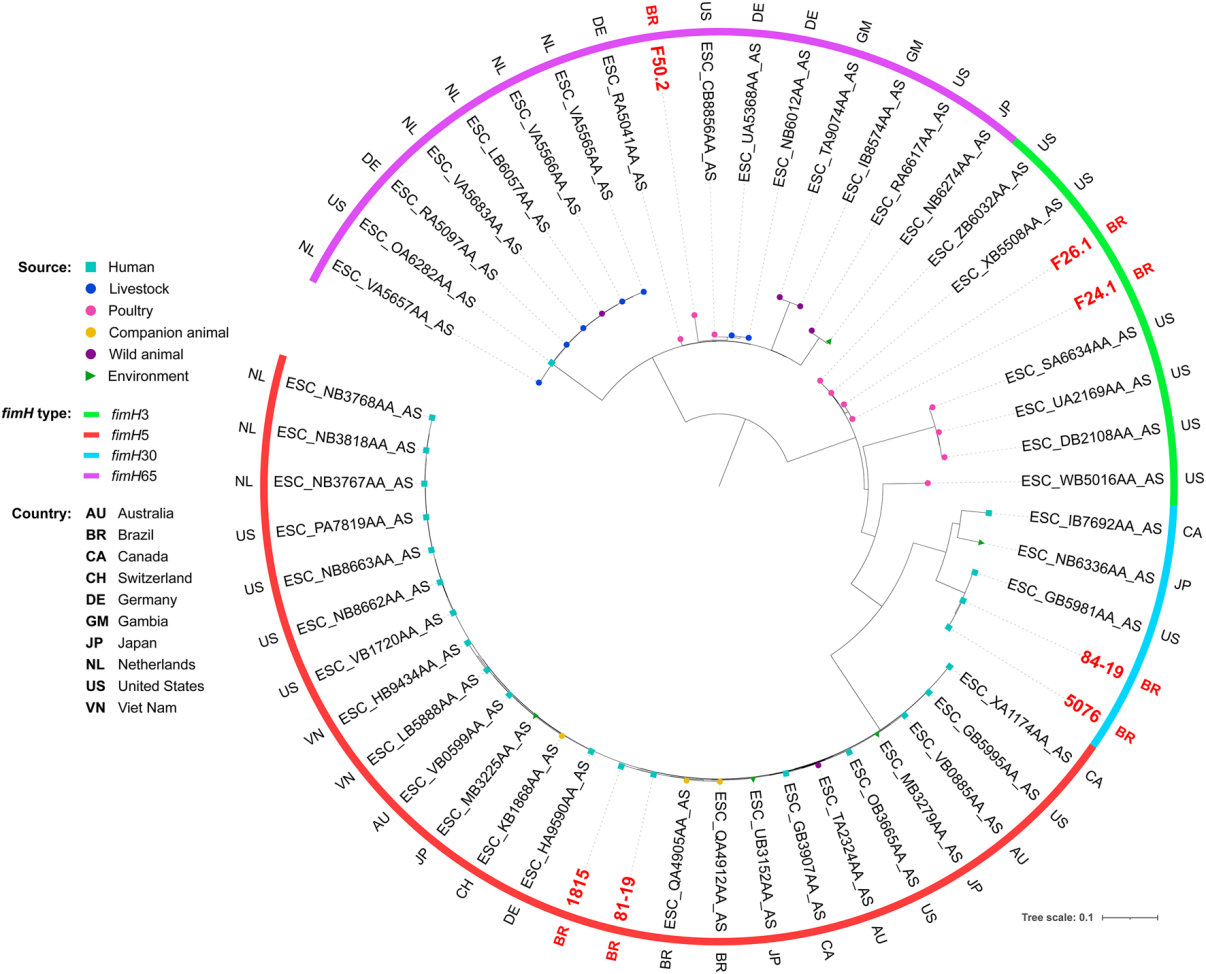
Phylogenetic tree of 54 *Escherichia coli* ST38 strains, their source, country of collection and *fimH* type.

**Figure 3:**
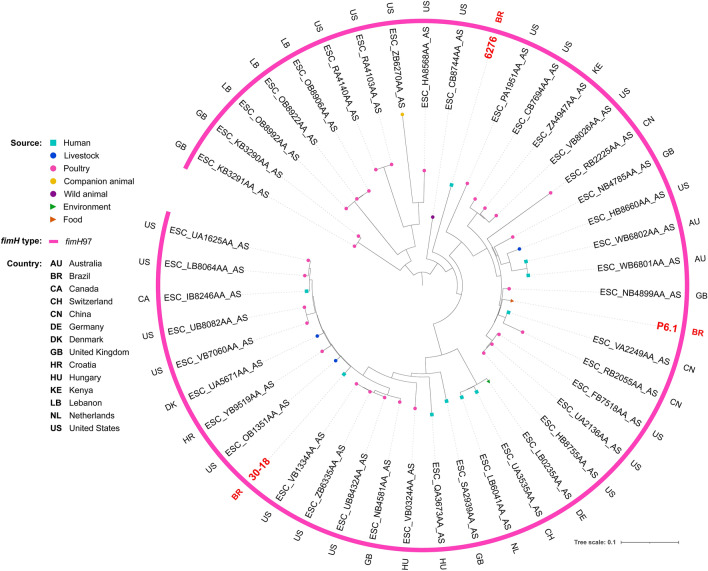
Phylogenetic tree of 46 *Escherichia coli* ST117 strains, their source, country of collection and *fimH* type.

**Figure 4:**
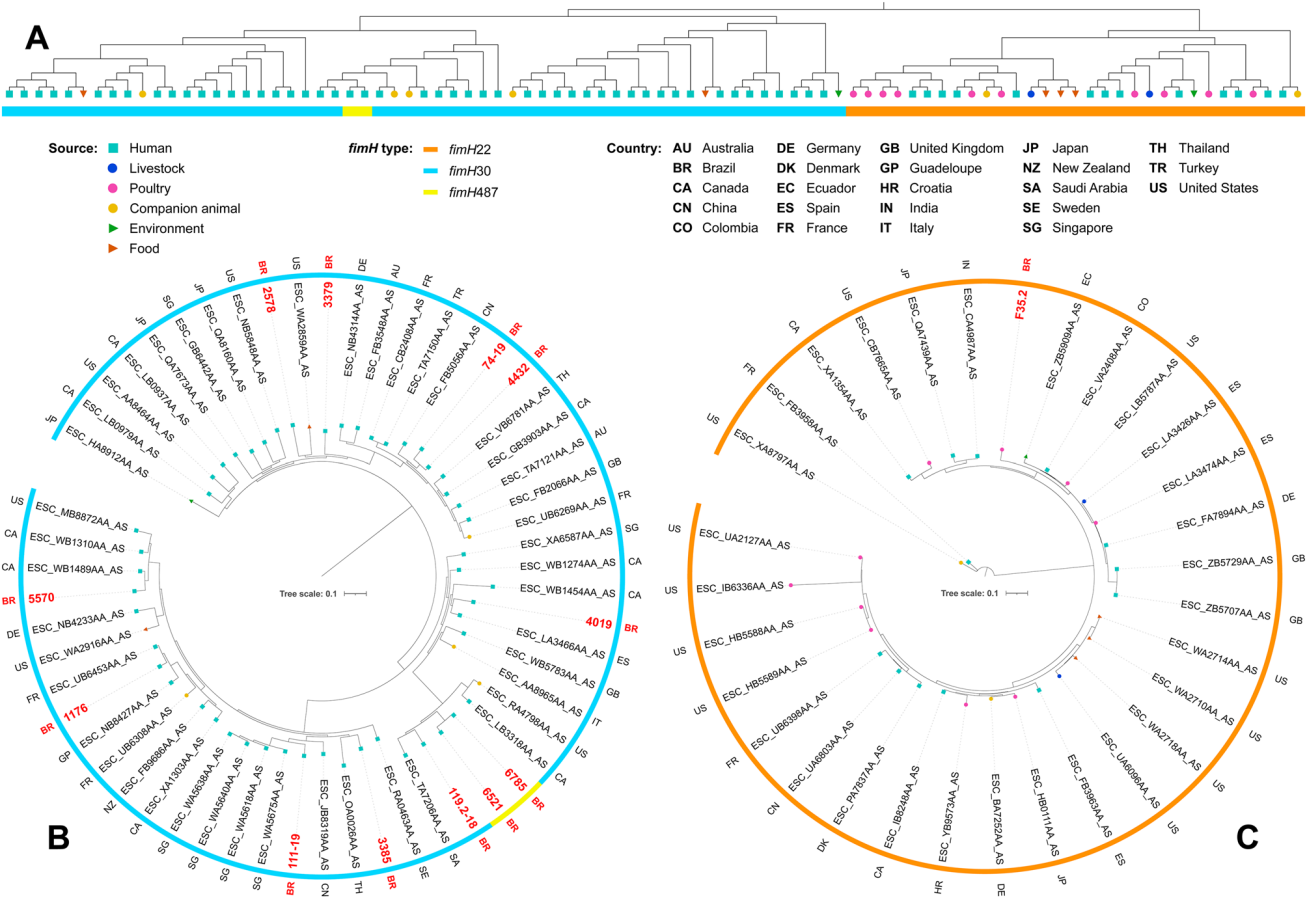
(**A**) Unscaled phylogenetic tree with the source and *fimH* type of 88 *Escherichia coli* ST131strains. (**B**) Subtree containing *fimH*30 and *fimH*487 isolates showed in (**A**), their source, *fimH* type and country of collection. (**C**) Subtree containing *fimH*22 isolates showed in (**A**), their source, *fimH* type and country of collection.

**Figure 5:**
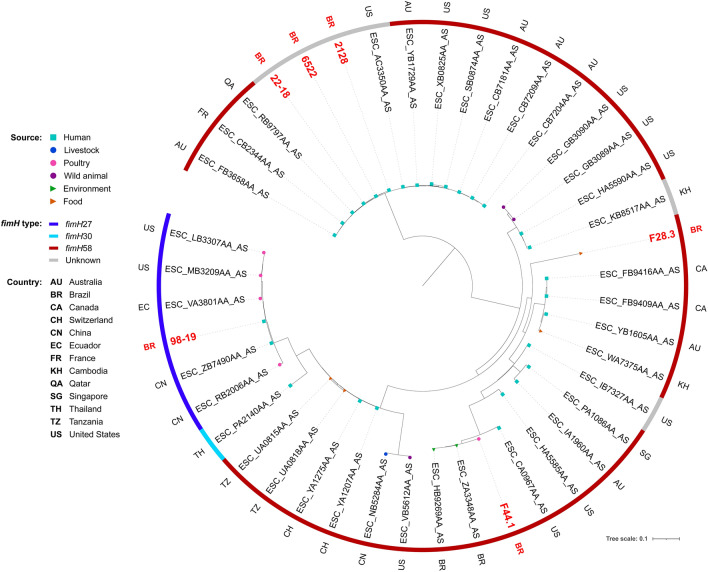
Phylogenetic tree of 43 *Escherichia coli* ST354 strains, their source, country of collection and *fimH* type.

**Figure 6:**
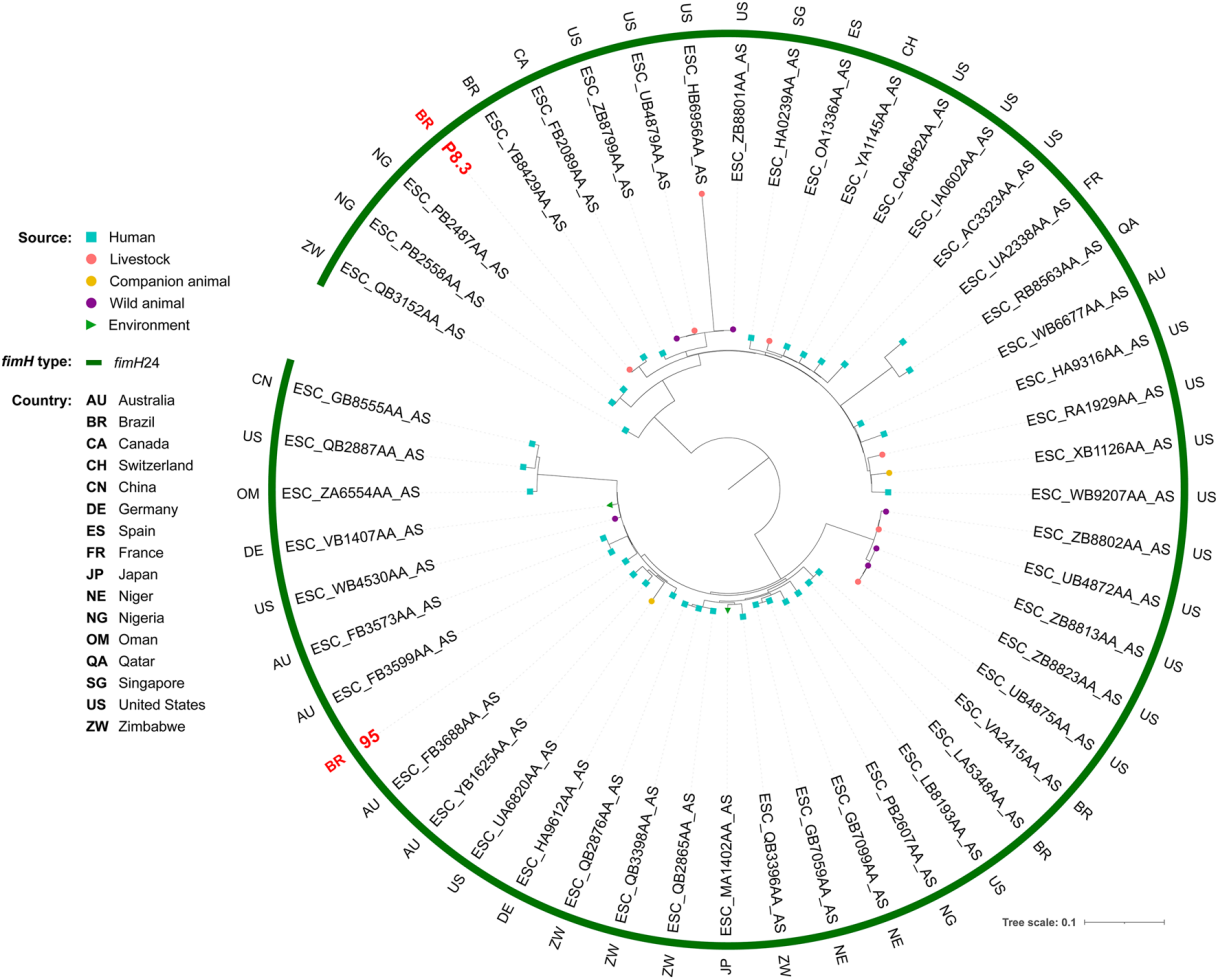
Phylogenetic tree of 51 *Escherichia coli* ST410 strains, their source, country of collection and *fimH* type.

**Figure 7:**
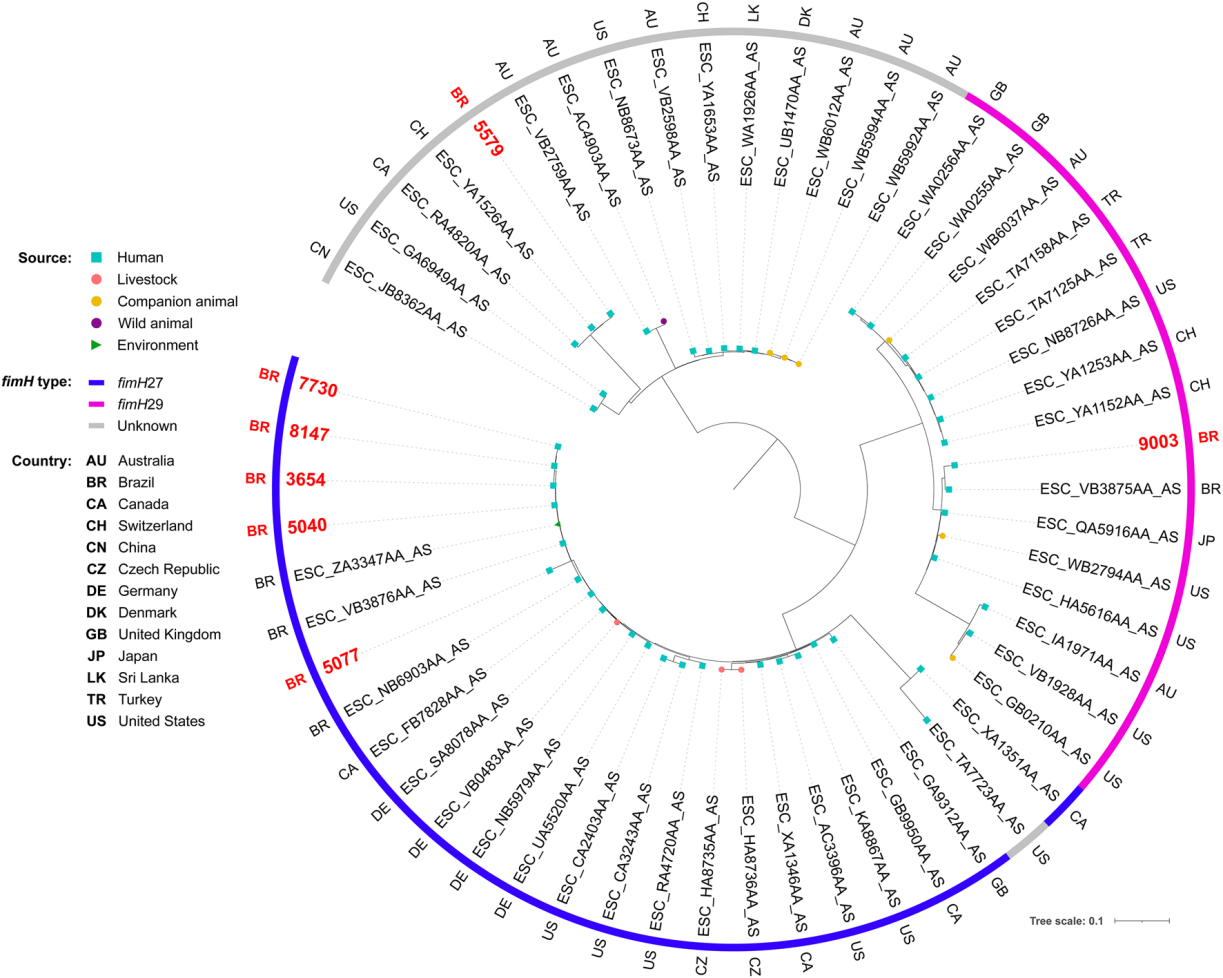
Phylogenetic tree of 56 *Escherichia coli* ST648 strains, their source, country of collection and *fimH* type.

Phylogenetic analysis revealed genomic diversity among isolates. In fact, most of our isolates of the same ST grouped within different clades, closer to isolates from several different countries. For ST38, isolates grouped within two different clades, both mainly composed by strains of human sources (Fig. 2). While two chicken isolates grouped together within a clade with only strains of poultry sources, the other grouped within a clade mainly composed by strains from livestock and wild animal origin. For ST117, most of the isolates were from poultry origin, including closely related strains from human host (Fig. 3). For ST131, while the strain from chicken grouped with strains from various animal sources, human isolates grouped mainly with other human isolates, but isolates were relatively distant to each other, being closer to strains from several other countries (Fig. 4). For the ST354, three of human isolates grouped together within a clade shared only with human isolates, whereas other human isolates clustered into a clade, mainly composed by poultry strains. The isolate from food grouped within a clade with human, poultry, environment and food isolates (Fig. 5). For ST410, the human isolate grouped within a clade shared mainly with strains from human sources, and the isolate from swine grouped within a more diverse clade (Fig. 6). For ST648, human isolates grouped within three different clades, all of them composed mainly by isolates from human origin (Fig. 7).

## Discussion

This study presents the first reports of *E. coli* ST131-C1-M27 in human infection and CTX-M-24-positive *E. coli* ST354 from UTI, in Brazil. In this country CTX-M-producing *E. coli* are endemic. Our data show a wide distribution of these isolates belonging to international clones in livestock and community patients. The extensive presence of CTX-M enzyme-producing strains in several sources raises the hypothesis that the spread occurs with greater frequency and efficiency, especially among Enterobacterales^10^.

*E. coli* ST131 is globally disseminated and related to the spread of resistance genes, including specific CTX-M variants^17^. Recent studies have shown that ST131 is rare among animal isolates, becoming almost exclusively a human pathogen, as demonstrated by our results, where ST131 was predominantly found a human lineage isolated from urine^18^. The *bla*_CTX-M-15_ gene is frequently found in *E. coli* ST131 UTI and is strongly related to the C2 subclade^11^. Different groups of plasmids can carry the *bla*_CTX-M-15_ gene, here we observe that this gene is involved with the incompatibility group IncF. In a previous study was shown that clade C was related to highest rates of UTI, with subclade C2 being the most common and associated with incompatibility group IncFII^19^. Besides, CTX-M-15-producing *E. coli* ST131 has already been shown to be involved in outbreaks in health institutions and is the most prevalent ESBL-producing *E. coli* worldwide^20^.

The CTX-M-27-producing ST131-C1 has been considered a new epidemic clone, and it has not been reported in human infections so far, in Brazil. Clade C1-M27 is associated with CTX-M-27 and was first observed colonizing children in France in 2012. Birgy et al*.* (2019)^21^ suggests that the C1-27 subclade was recently emerged, because it was observed that the difference in SNPs between the C1 subclade isolates was smaller compared to the SNPs difference between the C2 subclade and the A clade isolates. In addition, the plasmid predominantly involved with the dissemination of *bla*_CTX-M-27_ is IncF[F1:A2:B20], as found in our study. Resistance to fluoroquinolones, macrolides, tetracyclines, aminoglycosides, and sulfonamides appears to be part of the profile of C1-M27 isolates^21,22^.

CTX-M-14 and CTX-M-24 ESBL variants belong to the CTX-M-9 group. Although the first one is widely distributed, especially in China, South-East Asia, Japan, South Korea, and Spain, microorganisms producing CTX-M-24 remain relatively rare, being reported with greater incidence in countries such as Peru and Bolivia^19,23,24^. This study found an association between CTX-M-24 and *E. coli* ST354 detected in two human isolates, never before reported in UTI in Brazil. Strains of ST354 isolates were positive to *bla*_CTX-M-24_ and displayed further resistance to ciprofloxacin, being associated with extra-intestinal infections, animals and humans, reinforcing the zooanthroponotic hypothesis of these clones^25^.

CTX-M-2 and CTX-M-55 ESBL variants are commonly reported^26^. The co-production of CTX-M-8 and CTX-M-55 is not a common phenomena, which was first described in poultry samples in Brazil, in 2018^12^. In this regard the *bla*_CTX-M-8_ gene has been located in plasmids IncI1, and the *bla*_CTX-M-55_ gene in plasmids IncF^12^, being confirmed by our results. Therefore, the coexistence of CTX-M-8 and CTX-M-55 in two of our human urine isolates belonging to ST1725 can be explained by the presence of plasmids IncF[F33:A-:B1] and IncI1[ST-113], whereas in the chicken meat isolate belonging to ST6448 we detected IncF[F24:A-:B73] and IncI1[ST-131]^23^. In the last ten years, IncI1-type plasmids have had a high spread, mainly in animal reservoirs. There are reports of *bla*_CTX-M-2_, *bla*_CTX-M-8_ and *bla*_CTX-M-55_ genes frequently found on IncI plasmids from *E. coli* isolated from chickens and pigs several countries, such as China, France, the United States of America, and the United Kingdom^27–29^.

The international clone ST117 was the only ST found in all our three sources studied. This clone is often associated with AMR and found in animals and human isolates, having a zoonotic profile^30–32^. *E. coli* ST117 isolates have been identified in pigs, with the *bla*_CTX-M-55_ gene being carried by IncN-types plasmids. These data found in our study raises concern, as they may indicate a possible new epidemic transmission of CTX-M-55 in Brazil^31,33^.

ST38 was found in chicken meat and urine samples. Considered a minority among ESBL-producing *E. coli* in Europe, Middle East and Asia, the ST38 *E. coli* has been previously associated with *bla*_CTX-M-2_, *bla*_CTX-M-9_ and *bla*_CTX-M-14_ genes, but rarely with the *bla*_CTX-M-27_ gene^34,35^. The *bla*_CTX-M-27_ gene has been associated with IncF plasmids, with the most commonly identified being IncF[F-:A-:B33]^35^. Interestingly, in our study a ST38 *E. coli* isolate from human urine carried the *bla*_CTX-M-27_ and had plasmid IncF[F2:A-:B10]. Furthermore, recent reports linked CTX-M-types producing *E. coli* ST38 to UTI^36^. These data may indicate an increased frequency with which ST38 isolates are being found, being a prominent ESBL lineage worldwide^34–36^.

In summary, *E. coli* carrying *bla*_CTX-M_ genes from different sources seem to be related to the spread of internationally known clones (ST38, ST117, ST131, ST354, ST410, ST648). Some clones associated with CTX-M variants are more prevalent in some sources than others, which does not exclude the possibility that new clones are entering and establishing themselves in different niches. In fact, we detected *E. coli* ST131-C1-M27 in human infections, and CTX-M-24 positive *E. coli* ST354 from UTI, which could be a public health issue. Furthermore, the presence of CTX-M (-55, -27, -24, -15, -14 and -2)-producing *E. coli* among ST38, ST117, ST131 and ST354 clones from humans and meat for consumption reinforces the need for additional surveillance studies, in order to study dynamics of dissemination of ESBL-producing *E. coli* clones at the human-animal-food-environmental interface.

## Material and methods

### *E. coli* isolated from human urine samples

During June 2016 to May 2019, 195,080 urine cultures were performed in a public health services, in a city in south of Brazil. A total of 34,293 (17.6%) were positive for Gram-positive or Gram-negative microorganisms; of these 22,698 (66.2%) were *E. coli* strains and a total of 2033 (6.2%) ESBL producing bacteria, being 1389 (51.2%) ESBL-producing-*E. coli*.

As screening criteria for performing the ERIC-PCR, we selected 1,389 *E. coli* isolates from human urine that presented ESBL production and/or were resistant to quinolones, previously identified by the automated VITEK^Ⓡ^ 2 system.

Through the analysis of the dendrogram constructed by the ERIC-PCR technique, we selected 59 *E. coli* isolates from human urine that presented an interesting genetic similarity profile to perform the complete genome sequencing.

All participants provided the written informed consent for this study and the study was approved by the Ethics and Research Committee of the State University of Londrina by a document numbered as CAAE 56869816.0.0000.5231. Besides, we confirm that all experiments were performed in accordance with ethical regulations.

### *E. coli* isolated from chicken meat and pork samples

A surveillance study from January to May 2019 was carried out, to research ESBL-producing *E. coli*, in chicken and pork meat, bought at markets and butcher shop near public health services. Quinolone-resistant and ESBL-producing *E. coli* were investigated in chicken meat (n = 50), and pork (n = 50) samples. A total of 102 *E. coli* was isolated from chicken meat marketed, with 52 ESBL positive. And 67 resistant *E. coli* were isolated in pork meat, 31 ESBL positive.

We performed ERIC-PCR on all 169 *E. coli* isolates derived from chicken meat and pork that showed ESBL production and/or quinolone resistance. Through dendrogram analysis, we selected 32 *E. coli* isolates from chicken meat (n = 24) and pork (n = 8) that presented an interesting genetic similarity profile to perform the complete genome sequencing.

### Study population

The study relies on the complete sequencing of 91 *E. coli* isolates derived from human urine (n = 59), chicken meat (n = 24) and pork (n = 8).

### Microbiological methods

All the 195,080 urines collected from women patients during June 2016 to May 2019 was inoculated on CHROMagar (Becton Dickinson, Heidelberg, Germany) and MacConkey (Merck, Darmstadt, Germany) plates using a calibrated inoculating loop with a capacity of 10 μl and incubated at 37 °C for 24 h.

The samples of chicken meat (n = 50) and pork (n = 50) were dipped in Brain Heart Infusion (BHI) broth (Oxoid) with cefotaxime (4 µg/mL), ciprofloxacin (4 µg/mL), and both (Sigma-Aldrich, Munich, Germany) to selected resistant *E. coli* strains. After incubation, the solution was inoculated in the same way used for urine samples. We evaluated the growth of *E. coli* from the three BHI solutions and we preferentially selected the isolates that grew in broth with both antimicrobials (cefotaxime and ciprofloxacin). In case there was no growth of any isolate on that broth, we selected from the broth containing separate cefotaxime and ciprofloxacin. All the isolates selected were stored in Tryptic Soy Broth (TSB) with 15% glycerol (− 20◦C).

The identification and bacterial susceptibility were performed by the automated VITEK^Ⓡ^ 2 system, using the VITEK^Ⓡ^ 2 AST 239 card and the VITEK^Ⓡ^ 2 GN ID card (BioMérieux, USA). The bacterial susceptibility was tested for 14 antibiotics: ampicillin, amoxicillin/clavulanate, ceftriaxone, cefepime, ertapenem, meropenem, nalidixic acid, ciprofloxacin, norfloxacin, gentamicin, amikacin, nitrofurantoin, trimethoprim-sulfamethoxazole, and piperacillin-tazobactam. The CLSI 2020 (Clinical and Laboratory Standards Institute) criteria were used for interpretation. *E. coli* ATCC^Ⓡ^25922 strain was used as quality control.

All the time of study, samples were phenotypically confirmed for ESBL production using the chromogenic agar medium (ChromID ESBL bioMèrieux, Marcy L’Etoile, France).

### ERIC-PCR

1389 ESBL-producing isolates were subjected to Enterobacterial Repetitive Intergenic Consensus (ERIC-PCR), by Versalovicet al. (1991)^37^. Analysis of genomic fingerprinting was performed using GelJ v.2.0 software by the Dice similarity method (HERAS et al., 2015)^38^. Strains were considered genetically related if the similarity index was ≥ 85%.

### DNA isolation and whole-genome sequencing

For DNA extraction, strains were grown on Mueller–Hinton Agar overnight at 37 °C. Subsequently, a single colony was inoculated in 2 mL of Luria–Bertani broth for 12 h at 37 °C. The suspension was used to continue extraction and purification by the DNA extraction kit (Invitrogen, Carlsbad, CA). The extracted DNA was quantified by Qubit dsDNA (double-stranded DNA) BR assay kit (Invitrogen, Carlsbad, CA). After quantification, the DNA was used to construct a paired-end library (150 bp), sequenced using the NextSeq platform (Illumina). The instructions of each manufacturer were followed in all steps.

### Bioinformatic analysis

Genome quality filter and assemblies were performed by the CLC Genomics Workbench version 7.0 (Aarhus, Denmark). Multilocus sequence type (MLST), resistome, and virulome were identified using MLST v2.0 (Larsen et al., 2012), ResFinder v3.1(Bortolaia et al., 2020), VirulenceFinder v2.0, (Joensen et al., 2014), PlasmidFinder v2.1 (Carattoli et al., 2014), FimTyper v1.0 (Roer et al., 2017) and SerotypeFinder v.2.0 (Joensen et al., 2015), respectively. The BacMet database (Pal et al., 2013) was used to identify biocides and heavy metal (HM)^32,39–45^.

In order to compare our strains belonging to ST38, ST117, ST131, ST354, ST410 and ST648 with other genetically related strains from different sources and countries, we performed a search for each ST on the Enterobase *Escherichia*/*Shigella* database^46^ (https://enterobase.warwick.ac.uk), then we downloaded all genome assemblies with data for country, source and collection year. Selection of assemblies to phylogenetic analysis was based on fimH type, obtained from Enterobase, and on average nucleotide identity (ANI), assessed with FastANI v1.32^47^ (https://github.com/ParBLiSS/FastANI).

CSI Phylogeny v1.4^48^ (https://cge.cbs.dtu.dk/services/CSIPhylogeny) was used with default settings to generate phylogenetic trees for each ST, using as reference genome the chromosome sequences of *E. coli* ST38 strain 144 (RefSeq accession numberNZ_CP023364.1), ST117 strain 14EC020 (NZ_CP024138.1), ST131 strain p4A (NZ_CP049085.2), ST354 strain SMS-3-5 (NC_010498.1), ST410 strain Ecol_517 (NZ_CP018965.1) and ST648 strain Ecol_881 (NZ_CP019029.1).

Phylogenetic trees were rooted at midpoint and annotated with data from Enterobase using iTOL v6 (https://itol.embl.de). Due to the great diversity of ST131, the tree was split into two subtrees, one for *fimH*22 and the other for *fimH30* and *fimH*485.

### Ethical approval

The study was approved by the Ethics and Research Committee of the State University of Londrina CAAE 56869816.0.000.5231.

## Supplementary Information

Supplementary Information 1.

Supplementary Information 2.

Supplementary Information 3.

Supplementary Information 4.

Supplementary Information 5.

Supplementary Information 6.

Supplementary Table 1.

## Data Availability

Draft whole-genome assemblies were deposited in GenBank under the BioProjectPRJNA578368. The data of the figures can be accessed in Figshare (https://doi.org/10.6084/m9.figshare.12808439.v1).
